# Antimicrobial resistance in enteric bacteria: current state and next-generation solutions

**DOI:** 10.1080/19490976.2020.1799654

**Published:** 2020-08-10

**Authors:** M. J. Wallace, S. R. S. Fishbein, G. Dantas

**Affiliations:** a Department of Pathology & Immunology, Division of Laboratory and Genomic Medicine, Washington University School of Medicine in St. Louis, St. Louis, MO, USA; b The Edison Family Center for Genome Sciences and Systems Biology, Washington University School of Medicine in St. Louis, St. Louis, MO, USA; c Department of Molecular Microbiology, Washington University School of Medicine in St. Louis, St. Louis, MO, USA; d Department of Biomedical Engineering, Washington University in St. Louis, St. Louis, MO, USA

**Keywords:** Antimicrobial resistance, enteric pathogen, pathobiont, mobile genetic element, high-throughput sequencing, whole-genome sequencing, metagenomics

## Abstract

Antimicrobial resistance is one of the largest threats to global health and imposes substantial burdens in terms of morbidity, mortality, and economic costs. The gut is a key conduit for the genesis and spread of antimicrobial resistance in enteric bacterial pathogens. Distinct bacterial species that cause enteric disease can exist as invasive enteropathogens that immediately evoke gastrointestinal distress, or pathobionts that can arise from established bacterial commensals to inflict dysbiosis and disease. Furthermore, various environmental reservoirs and stressors facilitate the evolution and transmission of resistance. In this review, we present a comprehensive discussion on circulating resistance profiles and gene mobilization strategies of the most problematic species of enteric bacterial pathogens. Importantly, we present emerging approaches toward surveillance of pathogens and their resistance elements as well as promising treatment strategies that can circumvent common resistance mechanisms.

## Introduction

Antimicrobial resistance (AMR) is one of the most formidable threats to global human health. A recent report by the Centers for Disease Control and Prevention (CDC) declared that humankind has entered the dreaded “post-antibiotic era,” wherein we face infections resistant to every available treatment option.^1^ The transmission and spread of multi-drug resistant organisms (MDROs) is facilitated by astronomical increases in human travel and trade within the last few decades.^2^ Without adequate intervention, global death rates attributable to AMR are projected to surpass that of cancer and reach 10 million deaths per year by 2050.^3^ Nearly all of the most concerning pathogenic species associated with AMR spend some portion of their lifecycle within the mammalian gut.^1^^,2^

The human gastrointestinal (GI) tract contains a highly structured community poised with the potential to grow and transmit MDROs, with the gut microbiome containing an estimated 10^1^^4^ microorganisms.^5^ This multi-species ‘organ’ exists in open contact with external factors such as antibiotics and outside organisms that facilitate AMR development and spread. A considerable number of these factors are a product of the past century of human history, during which antimicrobial therapies were discovered and put into wide-scale use for treatment of human infections, animal husbandry, and agriculture.^6^ AMR is particularly challenging in the realm of hospital-acquired infection, where vulnerable populations are easily colonized by MDROs.^1^ Even before modern history, life in microbial communities has allowed bacteria to evolve with xenobiotic stressors, natural products or antibiotics, produced by competing microorganisms, resulting in an innate, if not “ancient,” resistome.^7^

This review focuses on AMR in enteric bacteria, as they are a significant cause of human infection and the human gut serves as a major conduit for the genesis and environmental spread of MDROs. Here we highlight major bacterial species that cause enteric disease, the AMR mechanisms they employ, and the various modes of AMR mobilization. It is important to note that while this review focuses exclusively on bacterial pathogens, there are extensive enteric morbidities and drug resistance associated with viral, protozoal, and fungal microorganisms, which have been reviewed elsewhere.^8–11^ In the context of enteric bacterial pathogens, we additionally suggest future avenues for prevention and treatment of enteric MDROs. In recent years, genomics- and metagenomics-based methods are increasingly being employed to survey circulating antibiotic resistance genes (ARGs) and predict diverse ARG mobilization strategies.^12^ A number of alternative solutions to standard antibiotics, in the form of vaccines, alternative antimicrobial targets, or probiotic cocktails, may provide some hope for the mitigation of AMR in the context of enteric disease.^13^

## Part I. Bacterial enemies, foreign and domestic

Within this review, the major enteropathogenic bacterial species are bifurcated into two groups: (1) invasive enteropathogens, which originate from an outside environmental reservoir, and (2) pathobionts, which originate from commensal gut species (Table 1). There are, of course, well-documented deviations from these delineations. For example, we categorize *Clostridioides difficile* as an invasive enteropathogen, yet it can colonize some hosts asymptomatically.^46^ Conversely, we classify *Escherichia coli* as a pathobiont, although some pathotypes of *E. coli* (discussed further below) are obligate invasive enteropathogens.^4^ The “switch” between these two categories can often be achieved rapidly through horizontal gene transfer (HGT) of genetic elements such as pathogenicity islands,^47^ which is discussed in Part II. It is important to note that across these pathogens, there are a range of *ad hoc* clinical diagnostic standards for determining susceptibility,^48^ and these practices are often absent or underutilized in the case of anaerobic pathogens such as *Clostridioides* or *Bacteroides* species.^49^

**Table 1.** Emerging and notable resistance mechanisms among enteric bacterial pathogens.

Species	Mechanism	Resistance Conferred	Reference
Invasive Enteropathogens			
*Campylobacter* species	Enhanced CmeABC multidrug efflux pump	Macrolides, fluoroquinolones, tetracyclines	14,15
	QRDR target site mutations in *gyrA*	Fluoroquinolones	16
	Ribosomal target site modification	Macrolides	17
	*ermB*-mediated methylation of 23S rRNA	MLS_B_ family, linezolid	14,18,19
	MDR genomic islands	Aminoglycosides, macrolides, fluoroquinolones	14,20
*Shigella* species	QRDR target site mutations and plasmid-mediated *qnrS*	Fluoroquinolones	21
	Overexpression of *acrA* efflux pump gene and plasmid-mediated enhanced efflux through QepAB and OqxAB	Fluoroquinolones	21–23
	IncF plasmid carriage of *mphA* or *erm* genes	Macrolides	24
	ESBLs including CTX-M, AmpC, and OXA	β-lactams	21
*Salmonella enterica*	Chromosomal QRDR mutations and plasmid-mediated *qnr* variants	Fluoroquinolones	25
	Plasmid-mediated *oqxAB* and *qepA*	Fluoroquinolones, aminoglycosides, and β-lactams	25
	Salmonella genomic island 1 encoding ACSSuT	Ampicillin, florfenicol, florfenicol, streptomycin, chloramphenicol, spectinomycin, antifolates, and tetracycline	26
	Plasmid-mediated mobile colistin resistance genes	Colistin	27
	Plasmid-mediated *blaCMY* genes	Cephalosporins	25
*Vibrio cholerae*	*vex* RND family efflux pumps	Erythromycin, novobiocin, penicillins, and polymyxin B	28
	SXT elements	Antifolates, streptomycin, nalidixic acid, tetracycline, and others	29
	QRDR target site mutations	Fluoroquinolones	30
*Clostridioides difficile*	Tn5398, Tn6194, Tn6215, or Tn916 transfer of *ermB* or *cfr* genes	MLS_B_ family and linezolid	31
	Metabolic pathway alterations such as DNA repair and iron uptake	Metronidazole	32
	Changes in peptidoglycan biosynthesis pathway, likely MurG	Vancomycin	31
	Mutations in *rpoB*	Fidaxomicin, rifamycin	33,34
Pathobionts			
BFG species	Insertion sequence activation of genes	Carbapenem, β-lactam, metronidazole, and macrolides	35
	Conjugative transposons activated by two-component regulatory system, most notably CTnDOT	Tetracyclines and erythromycin	36
	*bmeABC* efflux pump	β-lactams, carbapenems, cephems, metronidazole, quinolones, etc.	37
	*erm* gene-mediated methylation of 23S rRNA	MLS_B_ family	36
*Enterococcus faecalis* and *Enterococcus faecium*	van operon	Glycopeptides	38
	Target site mutations in LiaFSR, *gpdD, cls*, YycFG	Daptomycin	39
	Target mutation or methylation of 23S rRNA	Linezolid	40
*Escherichia coli*	AmpC β-lactamase	Cephalosporins	41
	Carbapenemases including NDM, KPC, and OXA	Carbapenems	42
	Upregulation of *acrAB*, QRDR point mutations	Fluoroquinolones	43
	Plasmid-mediated mobile colistin resistance genes (*mcr1-9*)	Colistin	44,45

QRDR, quinolone resistance determining region; MLS_B_, macrolides, lincosamides, streptogramins B; MDR, multi-drug resistance; ESBL, extended-spectrum β-lactamase; ACSSuT, resistance to ampicillin, chloramphenicol, streptomycin, sulfamethoxazole, tetracycline; RND, resistance-nodulation-division; NDM, New Delhi metallo-β-lactamase; KPC, *Klebsiella pneumoniae* carbapenemase; OXA, oxacillinase.

### Professional pathogens: the invasive enteropathogens

**Figure 1.**
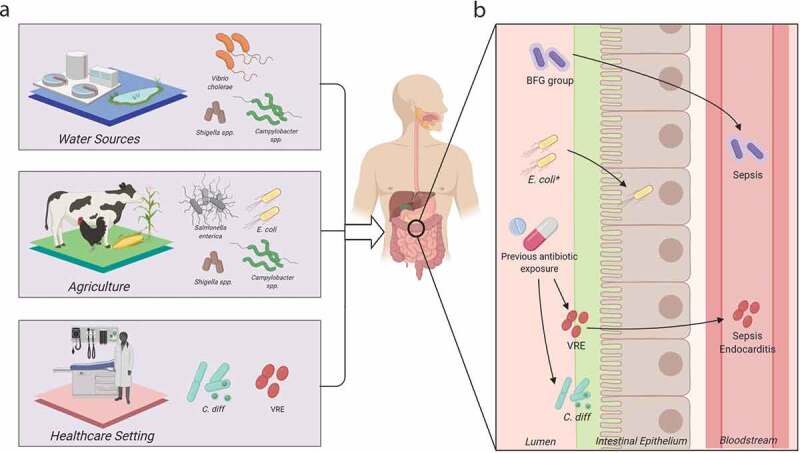
Major bacterial enteropathogens, antibiotic resistance reservoirs, and pathogenesis in the human gut. (a) The major enteric bacterial species and common reservoirs for proliferation and resistance exchange. (b) A close-up view of the human gut, representing various pathobiont species and pathogenic tendencies. *C. diff: Clostridioides difficile*, VRE: Vancomycin-resistant enterococcus, BFG: *Bacteroides fragilis* group. *Indicates *E. coli* can assume multiple pathogenic manifestations within the gut, as described by Kaper and coauthors.^4^ Image made with BioRender.

Invasive enteropathogens do not typically occupy the human microbiome as commensal species, and upon pathogenesis they can inflict acute intestinal distress including gastroenteritis, inflammation, and diarrhea. If not treated properly, extensive morbidities such as dehydration, bacteremia, shock, and even death may ensue.^50^ Diarrheal disease accounts for over 1.6 million deaths worldwide and is one of the top five causes of mortality for children under five.^51^ Many of these diseases are endemic to specific regions, but increased globalization has accelerated  international transmission of MDROs.^2^ Furthermore, common reservoirs of infection include water sources, food, and animals (Figure 1a).^2^ Invasive enteropathogens employ diverse mechanisms of AMR, which exacerbate the associated burdens on human health and the economy.^1^

#### Campylobacter species

*Campylobacter* species, including *C. jejuni* and *C. coli*, are a dominant cause of gastroenteritis and diarrhea, with rates of campylobacteriosis increasing worldwide.^52^ Acquisition of *Campylobacter* infection is often foodborne and linked to fecal contamination of water sources (Figure 1a); multiple animal reservoirs, most especially poultry,^53^ can host *Campylobacter* species. Campylobacteriosis is typically self-limiting, with empiric use of antibiotics such as fluoroquinolones in settings of acute disease.^50^ AMR in *Campylobacter* is highly prevalent in the United States, with over 400,000 cases of drug-resistant campylobacteriosis of the 1.5 million estimated total cases of infection.^1,14^ Resistance to both azithromycin, the drug of choice, and ciprofloxacin, a key second-line option, have appeared in multiple forms.^50,54^ Fluoroquinolone resistance can arise through point mutations in the quinolone-resistance-determining region (QRDR) of the fluoroquinolone target *gyrA*;^16^ this mechanism of resistance is conserved across many enteropathogens (Table 1). Within *Campylobacter*, QRDR mutations are synergistic with recently described, “enhanced” versions of the resistance-nodulation-division (RND) multi-drug resistance (MDR)-conferring efflux pump CmeABC.^15^ This efflux pump system has emerged with mutated regulatory regions that increase transcription of *cmeABC* and increase resistance to macrolides and fluoroquinolones (Table 1). It is likely that this efflux pump operon is controlled by multiple regulators, some of which may be drug-activated.^15,55^ Finally, AMR in *Campylobacter* species may be linked to the organism’s natural competency, allowing it to sample the community for transferable resistance.^56^ These observed MDR profiles and increasing rates of drug-resistant campylobacteriosis pose *Campylobacter* species as a serious global health threat.

#### Shigella species

*Shigella* bacteria are another major source of food poisoning and diarrheal disease.^1,54^ In 2016, shigellosis was the second leading cause of diarrheal death worldwide at over 200,000 deaths per year, ranking second only to rotavirus.^51^ The genus *Shigella* contains bacteria closely related to *E. coli* and is comprised of four major pathogenic species: *S. dysenteriae, S. flexneri, S. boydii*, and *S. sonnei*.^21^ *Shigella* can be transmitted from person-to-person, or through contaminated food sources and water (Figure 1a), while some AMR *Shigella* outbreaks are associated with international travel and sexual transmission.^57^ *Shigella* infections were once highly responsive to cheaper antibiotics such as β-lactams and antifolates, but rising resistance rates have shifted the treatments of choice toward macrolides or fluoroquinolones, with ceftriaxone as an alternative treatment option.^50^ MDR *Shigella* can arise through plasmid-borne or integron-mobilized elements encoding multiple types of resistance.^21,54^ A commonly observed MDR phenotype includes resistance to ampicillin, chloramphenicol, streptomycin, sulfonamides, and tetracyclines (ACSSuT).^21,24,58^ Epidemics driven by MDR *Shigella* have risen worldwide within the last decade and requires significant intervention efforts to prevent further disease.

#### Salmonella enterica

Another enteropathogenic species within the Enterobacteriaceae family is *Salmonella enterica*. The serovars of *S. enterica* are divided into typhoidal and non-typhoidal *Salmonella* (NTS), and encompass a group of bacteria that occupy digestive tracts of both animals and humans (Figure 1a).^59^ Although borne from the same species, the clinical manifestations and the associated immune responses are distinct among serovars. The GI distress associated with typhoid fever is due to the typhoid toxin and the damage it inflicts upon the GI epithelium. Typhoid fever is almost always treated with antibiotics.^25,59^ This disease is more common to developing countries, while NTS is common to both developed and developing countries. NTS infections present with gastroenteritis and diarrhea.^59^ NTS is usually self-limiting, and antibiotics are typically avoided since they may induce prolonged shedding of infectious NTS after treatment.^59^ Inappropriate antibiotic use is a key driver of AMR in *Salmonella*, and resistant infections often worsen clinical outcomes.^1,25,60^ *Salmonella* is notorious for its genomic islands, including *Salmonella* genomic island 1 (SGI1) carrying the ACSSuT region, encoding MDR (Table 1).^26^ More recently, some NTS serovars have evolved a novel genomic island encoding streptomycin and azithromycin resistance, which is concerning given that azithromycin is a second-line agent.^61^ Since *Salmonella* continues to be a major source of enteric infection, it will likely continue to present severe human health burdens without serious interventions.

#### Vibrio cholerae

*Vibrio cholerae* is the causative agent of cholera, a diarrheal disease attributed to upwards of 120,000 deaths per year.^62^ As with many diarrheal diseases, antibiotics are only required in the case of severe infections. First-line therapy typically includes doxycycline, while azithromycin, ciprofloxacin, and ceftriaxone are alternative therapies.^50^ *Vibrio* achieves extensive antibiotic resistance through its natural competency, allowing it to take up mobile genetic elements (MGEs) including plasmids, integrons, conjugative transposons, and SXT elements (Table 1).^30,63,64^ SXT elements, named for early observations of their conferred resistance to sulfamethoxazole and trimethoprim, are a type of integrative and conjugative element (ICE) that can confer additional resistance to agents such as streptomycin, nalidixic acid, and tetracycline.^29^ Similar to other diarrheal pathogens, *V. cholerae* acquires QRDR mutations that result in fluoroquinolone resistance, and can acquire diverse classes of efflux pumps conferring resistance to agents such as erythromycin, penicillins, novobiocin, and polymyxin B (Table 1).^28^

Cholera outbreaks have recently arisen in many developing countries, with the largest recorded cholera outbreak occurring in Yemen.^65^ The continual and largely preventable cholera epidemics have been considered “the world’s worst humanitarian crisis” by the United Nations.^65^ Due to the aggressively large impact on global human health, diverse resistance mechanisms against frontline agents, and the potential for further spread of resistant infections, *V. cholerae* is one of the highest priority enteric bacterial pathogens.

#### Clostridioides (Clostridium) difficile

*Clostridioides difficile* is a leading source of hospital-acquired enteric infection, causing nearly a quarter million infections and over 12,000 deaths per year in the United States alone.^1^ Although we have classified *C. difficile* as an invasive enteropathogen, it can asymptomatically colonize the human gut.^13^ *C. difficile* infection (CDI) likely occurs because of a loss of host colonization resistance, due to risk factors and co-morbidities such as antibiotic exposure or chemotherapy.^66, 45^ *C. difficile* is transmitted and ingested as a metabolically inactive spore. The metabolic environment of a dysbiotic GI tract is thought to facilitate germination of spores, leading to the development of CDI.^67^ The capacity to exist in various metabolic states, including dormant spores and withinbiofilms, is thought to contribute to its innate resistance to a number of antibiotics and sterilizing agents.^31^ In a clinical setting, some of the most virulent *C. difficile* ribotypes are also the most phenotypically drug-resistant. The spectrum of virulence in *C. difficile* is enhanced by a mobile genome, where 11% of the core genome of *C. difficile* is made up of MGEs.^68,69^ These MGEs are primarily represented by conjugative transposons, which are known to harbor MDR (Table 1).^31^ Transposon-independent resistance to vancomycin, rifampin and others has also been documented.^68^ Fidaxomicin is a more recently approved treatment option and resistance to date is rare. However, resistance has already been observed through point mutations in *rpoB*, the β subunit of the RNA polymerase target.^33,34^ Given that susceptibility testing for anaerobes such as *C. difficile* is not standard practice in the clinic, the extent of AMR in circulating *C. difficile* strains may be underestimated.

### Breaking bad: the pathobionts

Pathogenic strains evolving from commensal species are known as “pathobionts.”^70^ Pathobionts are increasingly recognized as a significant source of infection and a key reservoir of AMR. This heightened prominence of pathobionts in today’s society can be largely attributed to advances in modern medicine over the last century. Although the human lifespan is longer than ever, as infectious disease has become less of a threat, extensive antibiotic use within people and the environment (Figure 1b) as well as a substantial rise in vulnerable populations has accommodated the rise of the pathobionts.^6^

#### Bacteroides fragilis *group (BFG) species*

The *Bacteroides* and *Parabacteroides* species within the BFG group include some of the most well-characterized commensal GI species, but are also the most commonly isolated organisms in anaerobic extraintestinal infections and increasingly reported to harbor AMR.^36^ Although the most ubiquitous resistance elements in BFG confer resistance to classes such as tetracyclines and macrolides, resistance to clinically useful agents such as β-lactams, carbapenems, and metronidazole is emerging in the United States and Europe.^35,71^ Resistance to all three treatment options can be achieved through “activation” of otherwise silent ARGs by insertion sequences.^35^ Conjugative transposons, most notably CTnDOT, have been well described among BFG species and commonly confer resistance elements against tetracyclines and erythromycin (Table 1).^36^ Clindamycin resistance has also steadily risen among BFG and is associated with acquisition of erythromycin resistance methylase (*erm*) genes that originate in gram-positive species.^36^ AMR only further exacerbates the morbidity and mortality rates associated with anaerobic infection, and further research into resistance mechanisms and prevention measures are desperately needed. Notably, susceptibility testing of anaerobes is not routinely performed in the clinic despite these emerging issues. Future efforts to tailor antibiotic stewardship should include emphasis on novel diagnostics for resistance in BFG and other anaerobes.^49^

#### Enterococci

*E. faecalis* and *E. faecium* are dominant causes of gram-positive nosocomial infections worldwide.^72^ Although Enterococci are well-established commensal species in the GI tract, Enterococci also cause extraintestinal infections, including endocarditis and sepsis (Figure 1b).^72^ Recent epidemics of vancomycin-resistant enterococci (VRE) have caused distinct clinical challenges in finding effective antibiotic regimens. After antibiotic use, enterococcal overgrowth in the GI tract creates an important reservoir for AMR development.^72^ Vancomycin resistance is acquired through the presence of *vanA* and *vanB* operons, which encode inducible synthesis of cell-wall modifications that reduce interactions between vancomycin and the cell wall.^73^ Additionally, these resistance loci often exist in a plasmidic element, or are associated with a transposable element, increasing the epidemiological threat of vancomycin resistance.^74^ Daptomycin and linezolid, representing newer antibiotic agents, are key treatment options for VRE infection, but resistance mechanisms are apparent for both agents (Table 1).^39,40^ VRE is clearly a large public health threat, especially in nosocomial settings, and preventative strategies should be pursued to mitigate the rise of drug resistance in enterococci.

#### Escherichia coli

Like other commensals, one of the most well-defined benefits of *Escherichia coli* to the human host is its ability to inhibit colonization by exogenous gut pathogens.^75^ In addition to the basal level of AMR that *E. coli* may harbor as a ‘commensal,’^76^ certain *E. coli* strains are highly pathogenic and form at least six pathotypes capable of causing both GI and extra-intestinal disease.^4^ The specific course of antibiotic therapy for *E. coli* infection is often guided by pathotype and/or strain type.^50^ For example, in the case of Shiga toxin-producing *E. coli*, antibiotics are frequently avoided as they can exacerbate associated morbidities. In addition, among Enterobacteriaceae bacteria such as *Escherichia, Salmonella, Shigella, Enterobacter*, and *Klebsiella*, AMR elements of many classes are shared between these genera.^44,77-79^ Carbapenem-resistant Enterobacteriaceae (CRE) have been listed as an “urgent threat” by the CDC, while ESBL-producing Enterobacteriaceae have been categorized as a “serious threat”.^1^ The supposed agents of “last resort” include carbapenems and colistin, but as expected, resistance has arisen for these antibiotics over the last decade. Various β-lactamases and carbapenemases are increasingly documented among *E. coli* isolates (Table 1).^41,42^ *mcr* is an emerging ARG against colistin in Enterobacteriaceae, and at least nine *mcr* homologs (*mcr-1-9)* have been identified (Table 1).^45^ This gene encodes a phosphatidylethanolamine transferase that transfers a phosphatidylethanolamine molecule to lipid A within the gram-negative cell membrane, thus making the bacteria less susceptible to the action of colistin.^45^ Although *E. coli* is a proven commensal occupant of the human GI tract and a genetic workhorse in laboratory settings, it is responsible for highly-concerning MDR profiles worldwide and can represent a true “superbug.” Since resistance to virtually every clinically utilized antibiotic can be rapidly disseminated among pathogenic Enterobacteriaceae, it is critical to pursue preventative and alternative measures to treat these infections.

## Part II. Questionable arms deals: the evolution and acquisition of drug resistance

**Figure 2.**
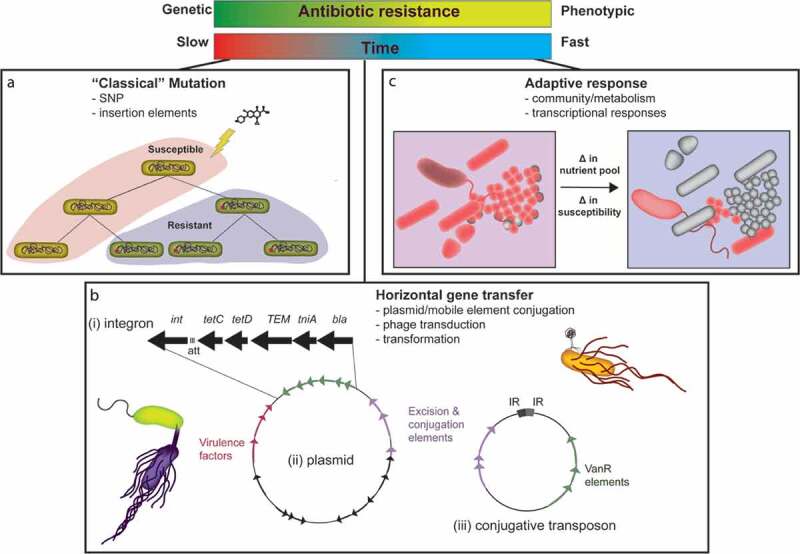
Manifestations of drug resistance. Bacteria become less susceptible to antibiotics through multiple mechanisms, from genetic acquisition of resistance to phenotypic responses to antibiotics. These mechanisms fall into three categories: classic mutation (a), horizontal gene transfer (b), and adaptive response (c). (a) Mutation via genome replication or intragenomic rearrangement can result in the acquisition of resistance. This process requires a series of bacterial generations to cause selection in the population for the inheritance of the resistance mutation. (b) Horizontal transfer of genetic material through multiple mechanisms in enteric pathogens, mainly bacterial conjugation, results in the more rapid acquisition of resistance elements. Mobile genetic elements such as integrons (i), plasmids (ii), or transposons (iii) can be transferred by conjugation. (c) An enteric pathogen’s susceptibility to antibiotics can be determined or altered based on the microbial community. In this example, a change in nutrient pools within a polymicrobial community can prompt a change in the inherent susceptibility of certain organisms to a given antibiotic. Often these susceptibility changes are mediated by changes in bacterial metabolism.

Commensal gut microbes and pathogens constantly evolve under the pressures of antibiotic exposure, whether in the gut or in an environmental reservoir. Depending on the bacteria’s ancestral lineage and the extent of allelopathy occurring in the microbe’s previous habitat, some bacteria are more intrinsically antibiotic-resistant.^7,80^ Here in Part II, we distill the diverse landscape of bacterial AMR mechanisms into a spectrum of antibiotic susceptibility, whereby a number of diverse pathways can lead to less susceptible organisms (Figure 2).

At one end of the spectrum are “classical” resistance mechanisms, including spontaneous mutations (Figure 2a) that modify target enzymes, alter transcription of select genes, or bypass antibiotic activity. In addition to nonsynonymous point mutations, these spontaneous mutations can also come in the form of insertion elements.^81^ Such evolved changes are inherited by the daughter cells through vertical transfer in subsequent generations.

Moving along the spectrum (Figure 2b), HGT is a phenomenon encouraged by the density of microbial communities both in the gut and in environmental reservoirs. We discuss movement of genetic elements in invasive enteropathogens and pathobionts through distinct examples of pathogen evolution (Figure 2b). HGT is positioned somewhere in the middle of the spectrum whereby bacteria acquire and shed genetic traits encoding resistance, often resulting in AMR acquisition more rapidly than spontaneous mutations.^47,82^

At the very end of the spectrum, bacteria are primed to respond to antibiotics through phenotypic variation, fluxes in bacterial metabolic state, programmed responses to the antibiotic/environment, or a systems-level effect of the bacterial community in the environment (Figure 2c).^83^ While these phenotypic states may not result in outright resistance, they provoke tolerance in the bacterial population and allow time for the organisms to acquire extensive AMR through other mechanisms.^84^ Genetic mobilization and adaptive strategies that result in clinically significant AMR are further described below.

### Major horizontal gene transfer mechanisms: pathogens sample their reservoir

Bacterial HGT occurs by conjugation, transformation, and phage transduction (Figure 2b). Conjugation is the most well-studied mechanism of HGT and likely the major contributor to AMR in enteric pathogens.^47^ Conjugation requires secretion machinery, either encoded on a plasmid or within the chromosome, to inject genetic material directly into a neighboring cell.^47^ The types of genetic material that undergo conjugal transfer are expanding and evolving. Conjugative plasmids, conjugative transposons, and integrative and conjugative elements (ICEs), often integrons, represent defined genetic units that can be transferred by conjugation.^85^ While these terms imply distinct DNA entities transferred by a single defined mechanism, the mechanisms of bacterial genetic mobility exhibit further complexity in that MGEs may contain other MGEs. (Figure 2b, panels i,iii).^85^ Conjugative plasmids contain elements mediating plasmid replication and transfer, and can also encode additional adaptive elements such as ARGs or virulence factors (Figure 2b, panel ii).^86^ Integrons contain an integrase gene and an *att* recombination site.^87^ Conjugative transposons contain sequence elements to excise, transfer, and integrate the entire ‘jumping’ fragment into a new genetic location.^88^ These elements can be as large as 65 kb, in the case of CTnDOT, a frequently transferred conjugative transposon carrying AMR in *Bacteriodes*.^36^ Given that enteric pathogens are under immense pressure to adapt to the GI environment and associated antibiotic exposures, new combinations of MGEs continue to emerge, as discussed below.

Rather than acquire DNA through conjugation, bacterial species can also acquire exogenous DNA through transformation, which is the direct uptake of DNA from the surrounding environment that has been excreted or released from lysed cells. It is well-established that bacterial competence is heavily dependent on the environment, which often conditions an organism’s competency.^89^ In the case of enteric pathogens, genera such as *Campylobacter* and *Vibrio* possess competence systems and can take up exogenous DNA that may confer resistance.^89,90^ While there is no evidence of transformation occurring *in vivo*, many of these pathogens are capable and poised to take up exogenous DNA in their environmental reservoirs.

A final and underappreciated mechanism of HGT is phage transduction, which has an emerging role in shaping the fitness and resistance of enteric pathogens. Phages are DNA or RNA viruses encapsulated by a protein coat or capsid, and replicate within a bacterial cell, possibly incorporating their genomes into the bacterial chromosome.^91^ There is controversial evidence of phage transduction of resistance *in vivo* and, additionally, multiple lines of evidence indicating that phages from multiple environments are circulating ARGs.^91–93^ Compelling and recent *in vivo* studies involving head-to-head competition of two *E. coli* strains in a mouse gut demonstrated that phage-mediated genetic exchange is responsible for adaptation of an invading *E. coli* species, underlining the likelihood of phage transduction as a mechanism of AMR spread.^94^ The proportion of mobilized AMR due to phage transduction in human enteric disease remains an open question.

### The mobilome of enteric pathogens: the ubiquity of mobility

Three concerning phenomena regarding MGEs are their mobility between environments and humans, their mobility across surprisingly unrelated bacterial taxa, and the potential for permanent integration of MGEs into the genome.^2^ These movements are captured in a growing appreciation for the “mobilome”, the group of all genetic elements that move within a chromosome, between chromosomes, and often, through plasmids. AMR acquisition via sampling of this mobilome often occurs in environmental niches that are external to the human host (Figure 1a). *Campylobacter* is found in multiple animal hosts and environmental niches, and thus has the opportunity to sample multiple ARG reservoirs. Remarkably, *C. jejuni* and *C. coli* can be isolated from livestock with phenotypic resistance profiles identical to those found in the clinic, especially in the case of fluoroquinolones.^16,95^ These ARGs appear to cluster on genomic islands or on circulating plasmids, indicating the frequent exchange of genetic material across environments or bacteria.^95,96^ In a subsample of *E. coli* isolates from both humans and poultry, a novel incompatibility group of plasmids has been discovered carrying a *bla*_CMY_ mobile element.^97^ In addition, multiple classes of integrons carrying AMR have been found in both commensal *E. coli* and *S. enterica* from ruminants.^98,99^ Future efforts should better characterize the spatiotemporal dynamics of these and other environmental MGEs as it relates to both human disease and sources of AMR in our agricultural and waste practices.

An alarming feature of HGT is that it can occur between a range of bacteria across species and even phylogenies, especially via conjugative elements. Perhaps the most appreciated exchange of resistance occurs within the Enterobacteriaceae. Within this group of bacteria, containing *Salmonella, Shigella*, and *Escherichia*, as many as 28 different plasmid types are circulating.^77^ Findings from a mouse model have recapitulated this interspecies transfer: in the mouse gut, a conjugative plasmid could move between *S. enterica* and a commensal *E. coli* species.^100^ Sequence-based analyses of ARGs found in certain pathogens indicates that elements may have been acquired from distant bacterial genera. For example, macrolide resistance elements, including *erm* genes, were identified in a number of animal-derived isolates of *Campylobacter*. Their closest gene homologs were found to originate from gram-positive genera.^101^ As previously described, enterococcal species can exchange *van* operons, encoding vancomycin resistance, via conjugative plasmids.^74,102^ Most evidence indicates that this conjugation occurs frequently within the genus, but there is *in vitro* evidence that *Enterococcus* spp. can conjugate with distantly related gut commensals such as *Lactococcus* spp. or *Bifidobacterium* spp.^103^ In the gram-positive pathogens, there is strong evidence that a Tn5397 conjugative transposon, encoding resistance to tetracycline, can move between *C. difficile* and *E. faecalis*.^104^ The extensive interspecies exchange of genetic information highlights the importance of systems-level approaches to identify patterns of ARG movement across diverse bacterial species.

The worrying outcome of the movement of such plasmids is not only the acquisition of resistance, but the potential for permanent deposition of mobile resistance elements into the pathogen’s chromosome. Perhaps one of the most notorious examples of this comes from ESBL plasmids, a family of conjugative plasmids responsible for MDR against multiple classes of β-lactams.^105^ β-lactamases of plasmid origin now appear to be chromosomally encoded in *Salmonella*^106^ and *E. coli* isolates (Figure 2b).^42,78^ In the gram-positive enteric pathogens, some *C. difficile* isolates have evidence of a ‘cryptic plasmid’ within their genome, an extrachromosomal piece of DNA that may be the result of a recombination event between a phage and plasmid.^69,107^ This genomic mobility within bacterial populations underlines the need to broaden our perspective on the factors that encourage the transition of AMR elements from the mobilome to the genome.

### Adaptive resistance: physiology engenders resistance

Phenotypic tolerance, or decreased susceptibility to antibiotics occurring independently of classical resistance mutations or mobilome-mediated acquisition of ARGs, can occur because of an innate cellular property or regulatory circuit present in the organism. Even in monoclonal *in vitro* studies, heterogeneity within a bacterial population, generated from stochastic properties such as permeability and gene expression, can lead to antibiotic tolerance, or decreased susceptibility in a subpopulation of the culture.^108–110^ Phase variation, wherein bacteria reversibly vary phenotypic properties within a population, can also reduce susceptibility.^111^

The addition of antibiotics or other stressors to a population results in bacterial adaptation which may be mediated through multiple forms of regulation, leading to antibiotic resistance and/or tolerance. For example, in the *E. cloacae* complex, a response-regulator complex senses change in cation concentrations and activates transcription of lipid A modification enzymes, causing a temporary increase in resistance to colistin.^112^ In Enterobacteriaceae, transcription of efflux pumps is often controlled by cellular sensing of antibiotics.^113^ In gram-positive bacteria, inducible macrolide resistance is regulated transcriptionally and post-transcriptionally, often upregulating programs to protect the ribosome or pump out the macrolide.^114^

The state of the bacterial population itself can prompt antibiotic tolerance and reduced susceptibility. In some distinct multicellular communities, such as bacterial biofilms, cells physically organize and form extracellular protective structures in a manner that promotes resistance for a subpopulation of cells or in the total population.^115^ In the case of Enterococci, they appear to form microcolonies in the gut which are biofilm-like communities. Within these communities, it is hypothesized that conjugative transfer of plasmids is greatly increased, possibly amplifying the resistance of the community.^116^ In *Campylobacter*, natural competency seems to be amplified in biofilms, facilitating the uptake of ARGs.^56^

More recently, a non-canonical form of altered drug susceptibility called heteroresistance has been characterized, wherein a subpopulation of a monoisolate culture survives and replicates in the presence of certain antibiotics while the remaining population is killed off. The resistance phenotype observed in heteroresistant cells is characteristically transient and reversible after removal of the antibiotic stressor, underlining an intimate link between heteroresistance and cellular physiology.^117^ In the case of *E. cloacae*, heteroresistance appears dependent on the presence of *phoQ*, a broad regulator of a number of cellular processes, including colistin resistance genes.^118,119^ *C. difficile* has also been described to have heteroresistance to metronidazole through an unknown mechanism.^120^ This phenomenon has been studied in other organisms and is not driven by one regulatory mechanism but rather, a number of diverse, stochastic cellular processes that are organism- and antibiotic-specific. Future efforts in continuing to understand, diagnose, and treat heteroresistance are greatly warranted. Furthermore, understanding the effect of the complex environment of the human gut on both innate drug susceptibility and phenotypic tolerance of enteric pathogens will be key to the development of novel therapeutic strategies.

## Part III. The way forward: harnessing next-generation methods to monitor AMR and effectively treat infections

Despite our recent foray into the dreaded post-antibiotic era,^1^ we are also entering a time of unprecedented technological advancement in biomedicine, genetics, and bioinformatics. These tools provide immense promise for improved AMR surveillance and diagnostics, as well as effective alternative strategies to treat and prevent AMR enteric infections. High-throughput sequencing (HTS) of both isolated species and metagenomic samples can be coupled with bioinformatics tools to predict and catalog ARGs as well as associated MGEs.^12,121^ In addition, we discuss alternative treatment options that circumvent traditional antibiotic therapies, which have encouraged resistance in the past.

### Predicting antibiotic resistance

Next-generation sequencing-based methods have increased capacities in the realm of pathogen surveillance and AMR profile characterization. These techniques can be performed on both isolate collections derived from the same species, or metagenomic samples representing mixed microbial communities, such as stool, soil, or wastewater. The promising utility and application of these genomics-based methods for the purposes of both pathogen surveillance and studying AMR has been thoroughly reviewed,^12,121^ and we present examples of how these methods have already provided incredibly useful insights into AMR surveillance, spread, and persistence in enteric bacteria. Furthermore, the potential for translating genomics techniques to clinical use is also discussed.

#### Comparative genomics of clinical isolates for MDRO surveillance

In recent years, sequencing-based methods have proven to be an invaluable tool for surveillance of enteric pathogen isolates and their corresponding resistomes. For instance, in the United States, the National Antimicrobial Resistance Monitoring System (NARMS) has used whole-genome sequencing (WGS) for the surveillance of *Campylobacter*^53^ and NTS.^122^ For both studies, hundreds of isolates were analyzed and showed a high degree of correlation between AMR genotype and culture-based resistance phenotypes. The genotype-phenotype correlation of *Campylobacter* ranged from 68–100% for identified ARGs, and based on one house-keeping gene, species-level resolution of the *Campylobacter* species could be achieved.^53^ The genotype-phenotype correlation was over 99% positive for NTS and revealed the first instance of ESBL carriage of *Salmonella* collected from retail meats in the United States.^122^

Another example of using comparative genomics to study isolate collections is a recent exploration of linezolid-resistant *E. faecium* isolates from the United States and Pakistan.^123^ Forty-nine draft genomes were constructed through Illumina WGS and 52 *E. faecium* genomes were obtained from public databases. The genetic mechanisms of linezolid resistance were distinct between the two geographic sites, with isolates from the USA having 23S rRNA mutations and Pakistan isolates having acquired ARGs including efflux pump genes and the chloramphenicol-florfenicol resistance (*cfr*) methyltransferase.^123^ Furthermore, MGEs associated with transposases and phage were proximal to the efflux gene *optrA*, suggesting HGT as a potential means of disseminating linezolid resistance. Disparate genetic mechanisms can therefore lead to similar resistance profiles among isolate cohorts. Clearly, WGS can uncover a comprehensive view of the multiple paths that lead to resistance and how they are mobilized.

#### Metagenomics-based methods to functionally characterize resistomes in the human gut

In addition to isolate collections, WGS-based methods are undoubtedly useful for phylogenetic and resistome analyses of metagenomic samples. In the case of functional metagenomics, HTS analyses of genomes can be coupled with culture-based approaches to infer the functional resistome of a metagenomic sample.^12^ This is especially useful in the case of novel resistance elements that are previously unannotated and likely not captured through standard bioinformatics-based approaches.

Neonates represent an especially vulnerable patient population, and therefore the characterization of pathogens and AMR within the neonatal gut microbiome is of intense interest. A recent study employed both shotgun sequencing metagenomic and functional metagenomics to characterize the gut microbiome and resistome of preterm infants who had spent their first few months of life in the neonatal intensive care unit.^124^ Analyses of fecal samples collected longitudinally during the first two years of life revealed that MDROs, including Enterobacteriaceae, are enriched after antibiotic exposure and persistently colonize the gut microbiota of neonates. Sequencing of metagenomic fragments identified as phenotypic resistance determinants through functional metagenomics revealed that the median identity of ARGs to a commonly used AMR database was only 32%.^124^ This highlights the discrepancy between currently available annotated resistance elements and the plethora of functional, circulating ARGs that are not identifiable by conventional genetic methods.

#### Intelligent methods to characterize mobilization and activation of AMR elements

As discussed in Part II, a key contribution to the true extent of drug resistance is not only the presence of ARGs, but also the ability to mobilize genetic elements in a manner that either transfers or activates their function. Computational tools to study these phenomena are increasingly being developed and improved; we describe a few examples of available tools in this section.^125^ Phasefinder is a recently developed tool to identify DNA-inversion events that result in phase variation, and has been used to identify invertible promoters upstream of ARGs in *Bacteriodes*.^111^ Site-specific, integrative MGEs can be detected using MGEfinder, which is especially suited for identifying transposable elements and can capture integration sites with apparent effects on AMR in both *in vitro* adaptive evolution experiments and in clinical isolates.^126^ HGT (or lateral gene transfer) events can be revealed with tools such as WAAFLE (http://huttenhower.sph.harvard.edu/waafle) or DarkHorse,^127^ and options are available for both isolates and metagenomes.^125^ Hi-C is a novel experimental approach that provides resolution on the association of plasmids with specific bacterial species, which is often lost during standard HTS practices.^128^ Long-read sequencing is also an emerging and increasingly cost-effective strategy to achieve better resolution on plasmid vs. chromosomal elements, and can assist with covering genomic sites that do not always attain adequate coverage such as repeated DNA elements at insertion sequence sites found upstream of the *cfiA* carbapenemase in BFG.^129^ These and other emerging technologies will likely prove essential in identifying elements beyond strict ARG sequences that can have a substantial impact on the extent of AMR in genomes or metagenomes.

#### Harnessing genomics for use in clinical microbiology

Genomics technology could be a beneficial tool for rapid and comprehensive resistance detection in clinical microbiology labs, yet certain hurdles remain before clinical implementation of these technologies. Current workflows can be time and cost-prohibitive, given the demand within a clinical setting. A recent study highlighted that genomics pipelines are highly variable between labs, with discordant results from the same sample depending on the ARG database and/or pipeline used, and further discordance with phenotypic resistance.^130^ Marrying WGS with phenotypic resistance is additionally complicated by the observation that phenotypic resistance can occur independently of the simple presence or mutation of one chromosomal ARG. Furthermore, functional metagenomics studies have revealed that a substantial fraction of genes that encode phenotypic resistance in clinical samples are not captured in current ARG repositories.^124,131^ Pinpointing ARGs to the causative pathogen in community samples can also be obscured by the fact that commensal bacteria often harbor innate or acquired ARGs.

Despite these obstacles, interest in the field and continual advances in genomics technology will likely spur this resource into routine clinical use in the near future. ARG repositories such as the Comprehensive Antibiotic Resistance Depository (CARD)^132^ and Resfinder^133^ are routinely updated and improved, and are therefore increasingly more reliable for comprehensive and accurate resistance detection. Advances in long-read sequencing technology, which can provide real-time diagnostic information, are enabling rapid, species-level resolution of ARG-harboring pathogens from metagenomics samples.^134^ A recent review suggests a simplified genomics pipeline for routine clinical use.^48^ Genomics technology has enormous potential for not only improving resistance detection in the clinic, but also for species identification, tracking virulence, and epidemiology.

### Treating the untreatable: alternative strategies for preventing and managing AMR enteric infections

It is commonly accepted that standard antibiotic therapies, once considered “miracle drugs,” are inadequate long-term solutions for drug-resistant infections.^1^ Resistance has been observed to virtually every clinically utilized agent, and even therapies with novel targets deployed in recent years have been rapidly met with resistance.^3^ The quandary of rapid resistance foiling the use of even the newest antimicrobial agents demands that alternative solutions be developed to manage the burden of enteric bacterial infections.

#### Vaccines

Vaccines are a promising strategy to mitigate infections without encouraging the spread of AMR. Alongside antibiotics, vaccines are one of the most life-saving innovations of the 20^th^ century. Vaccines can have long-term protective effects and harness the host immune system to clear pathogens before they can get a foothold on pathogenesis. Vaccines have already eradicated smallpox and nearly abolished polio infection.^135^ Licensed oral vaccines already exist for the two major enteric bacterial pathogens *Salmonella typhi* and *Vibrio cholerae*. Current research efforts toward *Shigella*, enterotoxigenic *E. coli* (ETEC), *Campylobacter*, and *S. paratyphi* are ongoing.^136^ Developing countries with endemic sources of enteric infection would stand to receive the most benefits from new vaccines.

#### Empowering the commensals

An emerging view is that gut dysbiosis, or a shift in the phylogenetic composition of the gut microbiome away from community compositions typically considered “healthy,” contributes to the success of enteric pathogens. This has inspired the development of modern therapeutic strategies to restore healthy microbiomes and therefore “empower” commensal populations in the gut to prevent pathogen colonization and proliferation. The healthy gut microbiota possesses metabolic functions that impair pathogen proliferation through direct nutrient competition or other indirect mechanisms.^137,138^ The path toward increased use of alternative therapies, such as probiotics and fecal microbiota transfer (FMT), can be forged by a deeper understanding of commensal-pathogen interactions and how AMR traits affect these interactions.^139^

Various efforts to harness beneficial bacteria to ameliorate enteric disease have been put forth in recent years. *Bifidobacterium* are a well-established component of the commensal gut microbiome and may provide protection against pathogen colonization early in life.^140^ *B. infantis* was given to a group of healthy term infants, resulting in a 90% decrease in ARGs in comparison to control infants.^141^ However, it is likely that the therapeutic solution to many states of dysbiosis will not be one bacteria, but rather, a community of bacteria. FMT may provide some efficacy in the case of recurrent CDI, as it may restore colonization resistance.^142^ Yet, a number of recent studies indicate that this procedure may have adverse outcomes due to transfer of possibly pathogenic, drug-resistant organisms.^143^ Rigorous screening of donor stools to avoid transfer of MDROs as well as efforts toward formulating a more defined “synthetic microbiota” may offer some hope toward avoiding MDRO transfer. In fact, for CDI, a number of synthetic cocktails of bacterial taxa have been discovered that appear to inhibit *C. difficile* proliferation in mouse models of disease.^144,145^ Further studies into the constituents of colonization resistance in a healthy human microbiota that provide protection against AMR enteric disease would spur progress toward this clinically promising approach.

#### Phage therapy

In addition to transporting genetic material between cells, phages have also exhibited potent antimicrobial activity against clinically important bacterial species.^13^ Phages can confer direct lytic effects on the target species or serve as payload carriers for antibacterial or antivirulence targets. Phages have high species specificity and have shown superior biofilm penetration to standard antibiotics.^146^ Although phage therapy is not currently an authorized therapy in Western countries, it has already shown promise as an experimental treatment in compassionate use programs.^13^ Phage therapy could serve as an ideal treatment for enteric pathogens, as phage treatment is not expected to encourage AMR and its specificity avoids damaging neighboring commensals.

#### Unusual targets and new combination therapies

Promising new treatment strategies in the preclinical pipeline include inhibitors against entirely new targets such as virulence factors and bacterial metabolic pathways that are absent in humans yet crucial for microbial survival, and targets that are highly implicated in adaptive resistance mechanisms such as persistence, tolerance, and heteroresistance. A recent review highlighted antimicrobial peptides and inhibitors of LpxC, which inhibit the first committed step in the biosynthesis of lipid A, as two inhibitor groups with broad interest in the field. Both inhibitor sets are especially promising for the treatment of gram-negative bacteria.^13^ Furthermore, antivirulence targets such as those that have well-established roles in persistence are of interest. New inhibitor series targeting the caseinolytic P protease system, an essential quality control process in many bacterial species such as VRE, can cause unchecked activation of the protease and nonspecific protein degradation, leading to cell death. These drugs seem especially effective in killing of stationary phase bacteria and persisters.^147^

The field has also developed a focus on other antibacterial strategies such as repurposing existing drugs, developing potentiators which enhance the activity of standard antibiotic therapies, or developing immunomodulators that harness the host immune system against the threat of infection.^13^ As an example, immunogenic activity derived from cholera toxin-conjugated siderophores protected mice from *Salmonella* infection.^148^ Furthermore, improved GI localization of metronidazole by conjugation to reutericyclin from *Lactobacillus* improved outcomes in a hamster model of CDI.^148,149^ It is important to continue to improve targeted delivery mechanisms to the GI tract such that collateral damage to the human and microbiome are minimized. Finally, combinations of existing antimicrobials have yielded surprisingly effective activity against recalcitrant pathogens such as heteroresistant bacteria. CRE clinical isolates (*Enterobacter, Escherichia*, and *Klebsiella*) display resistance to carbapenems but are sensitized in the case of treatment with multiple antibiotics.^117^ Rather than producing iterative homologs of existing antibiotics that may perpetuate existing AMR mechanisms, these alternative approaches offer promising and potentially life-saving options to mitigate enteric AMR infections.

## Concluding remarks

The definition of enteric pathogenesis has undergone considerable restructuring in the last several decades. A new appreciation for the complex relationship between the host microbiome, enteric pathogens, and AMR has shaped this understanding. AMR acquisition in the context of enteric disease appears to be a “systems-level” phenomenon in many cases, and is more complicated than the one gene, one bug explanation of AMR. Fortunately, the continual development of genomics-based approaches and bioinformatic tools is improving our understanding of what constitutes a healthy gut, an enteric infection, and drug resistance.^12,48^

Enteric bacterial pathogens will continue to exist, evolve, and present medical challenges to the human population. AMR is clearly an ever-present, innate, and ubiquitous feature of these pathogens that we must continue to strive toward understanding in order to formulate effective prevention and treatment strategies.^1,2^ In the lineup of pathogens discussed herein, we highlighted invasive enteropathogens and pathobionts, both of which cause life-threatening disease and are often recalcitrant to recommended treatment options because of wide-spread AMR. Within these organisms, we defined unique pathogenesis mechanisms, reservoirs of AMR transmission, and gene mobilization strategies. Furthermore, we presented the application and promising potential of next-generation surveillance, treatment, and diagnostic measures.

Although we presented several cutting-edge approaches to understanding and ameliorating the burden of AMR in enteric bacteria, other approaches beyond the scope of this review could have tremendous impact on alleviating the health burdens attributable to enteric disease. This includes changes in global health policies that will increase access to enteric disease treatment and prevention.^51,65^ Better management of antibiotic exposure in the environment and in the prevention of disease would undoubtedly reduce the spread of AMR worldwide.^3^
